# The impact of frailty on survival in elderly intensive care patients with COVID‑19: do not dismiss intensive care unit overcrowding

**DOI:** 10.1186/s13054-021-03653-y

**Published:** 2021-06-30

**Authors:** Romain Jouffroy, Benoît Vivien

**Affiliations:** 1 Intensive Care Unit, Ambroise Paré Hospital, Assistance Publique - Hôpitaux de Paris and Paris Saclay University, Boulogne Billancourt, France; 2 Service d’anesthésie réanimation, SAMU, Hopital Necker Enfants Malades, Assistance Publique - Hôpitaux de Paris, Paris, France; 3 Centre de recherche en Epidémiologie et Santé des Populations - U1018 INSERM - Paris Saclay University, Paris, France; 4 Institut de Recherche bioMédicale et d’Epidémiologie du Sport - EA7329, INSEP - Paris University, Paris, France; 5 SAMU de Paris, Service d’Anesthésie Réanimation, Hôpital Universitaire Necker - Enfants Malades, Assistance Publique - Hôpitaux de Paris, Paris University, Paris, France

**To the Editor:**

Recently in the Journal, Jung et al. [1] reported that frailty, age and comorbidities provide relevant prognostic information among elderly COVID-19 patients admitted to intensive care unit (ICU). While we fully agree with the authors claiming that the decision-making process relies on multi-components, COVID-19 pandemic, per se, implies cautions for the results interpretation. Firstly, the unprecedented influx of patients into hospitals and ICUs faced the physicians to a supplemental issue, the mismatch between means and resources [2, 3]. That was a crux in some low-income and middle-income countries (LMICs), at the origin of difficult daily triage decisions against the backdrop of severe shortages of basic equipment and consumables [4]. Secondly, the wide disparity for the reasons and the use of end-of-life care decisions due to cultural considerations could have induced a bias in this multicenter study from 28 countries. Thirdly, it would have been more representative to include in the analysis all frail patients: those younger than 70 years old, and those not admitted in ICU whatever the underlying reason [3, 5]. Last but not least, the relative weight of frailty, age and comorbidities covariates on outcome would be helpful to physicians in the day-by-day decision-making process, but the question is if one factor should be more important to consider than the others? Nevertheless, beyond all these limitations, we fully agree with Jung et al. that frailty assessment is one of the utmost important elements to take into account among COVID-19 patients, especially in elderlies [1].

## Data Availability

Not applicable.
